# Short follow-up evaluation of proximal femoral varus osteotomy for treatment of Legg–Calvé–Perthes disease

**DOI:** 10.1007/s10195-016-0412-0

**Published:** 2016-05-19

**Authors:** Mohamed Mansour Elzohairy

**Affiliations:** Faculty of Medicine, Zagazig University Hospitals, Zagazig, Egypt

**Keywords:** Proximal, Femoral, Varus, Osteotomy, Legg–Calvé–Perthes disease

## Abstract

**Background:**

There are many methods of treating Legg–Calvé–Perthes disease, including operative and nonoperative methods. Femoral varus osteotomy is one of the surgical methods used to treat this disease, and it involves changing the alignment of the proximal femur to improve containment of the femoral epiphysis in the acetabulum. The aims of this study were to evaluate the results of femoral varus osteotomy for the treatment of Perthes disease according to various classification and grading schemes, as well as to compare the results to those obtained using other methods of treatment reported in the literature.

**Materials and methods:**

Twenty-three patients with Legg–Calvé–Perthes disease were treated using a proximal femoral varus osteotomy procedure. The mean age of the patients was 7.8 years (range: 6–11.5 years). The average follow-up was 36.2 months (range: 29–48 months).

**Results:**

The patients were classified and graded according to the Catterall and Herring classifications. The preoperative and postoperative mean epiphyseal extrusion indices were as follows: group III (B), 10.88 % and 7.22 %, *P* = 0.027; group III (BC), 15.81 and 8.93 %, *P* = 0.005; group IV (C), 72.64 and 39.44 %, *P* = 0.018. The preoperative and the postoperative mean Wiberg’s CE angle were as follows: group III (B), 26.88° and 37.81°, *P* = 0.028; group III (BC), 24.4° and 32.2°, *P* = 0.005; group IV (C), 20.89° and 28.41°, *P* = 0.018. Changes in Iowa clinical hip scores were as follows: group III (B), 54.8 to 92.33, *P* = 0.027; group III (BC), 47.3 to 87.8, *P* = 0.005; group IV (C) 34.43 to 68.29, *P* = 0.017. In the last follow-up, the mean limb length discrepancy after plate removal was 0.9 cm (range: 0.0–2 cm) of shortening on the operated side. The author of the present study did not see any progressive change in this parameter during the follow-up period, especially after hardware removal and in the younger boys. All of the osteotomies united within 3 months without loss of fixation.

**Conclusion:**

According to the results of the present study, proximal femoral varus osteotomy gives good results in children between the ages of 6 and 10 years without any femoral head deformity and flattening, especially with good containment in abduction.

**Level of evidence:**

Level IV.

## Introduction

Legg–Calvé–Perthes disease (LCPD) is defined as an idiopathic osteonecrosis of the femoral head which leads to variable complications with resultant deformity of the femoral head and, later, osteoarthritis [26, 28]. Unlike normal, healthy bone, the bone of the avascular epiphysis is not capable of withstanding the stresses on the epiphysis of the femoral head in cases of LCPD. The aim of treating Perthes disease is to reduce the risk of later osteoarthritis by preventing femoral head deformity, which may occur if adequate containment is not achieved [11, 18, 19]. To achieve containment, the femoral head is centered within the acetabulum during the fragmentation and reossification phase. This allows the acetabulum to act as a mold during the healing or revascularization phase when the biologically plastic femoral head is at risk of subluxation, hinged abduction, and permanent femoral head deformation. At skeletal maturity, severe femoral head deformity and joint incongruity increase the risk of loss of function, leading to osteoarthritis later on [8, 17, 18].

There are many treatment methods for Legg–Calvé–Perthes disease, and the appropriate method to use depends on the grade of the disease. Such methods include spica cast immobilization, bed rest, traction, and walking with a weight-relieving caliper [3]. Surgical methods are also employed in young patients with Legg–Calvé–Perthes disease. Some authors have recommended nonoperative means such as bracing and cast immobilization, and have reported satisfactory outcomes for most patients [4, 9, 14]. Many other authors have reported good results with operative techniques such as femoral varus or valgus osteotomy, as well as other types of pelvic osteotomies such as innominate (Salter) pelvic osteotomy, lateral shelf osteotomy, and triple osteotomy [4–6, 8, 10, 14, 20, 35, 36, 39–41]. The study reported in the present paper was designed to investigate the short-term outcome results of proximal femoral varus osteotomy in the treatment of Catteral grade III (Herring groups B, BC) and IV (Herring group C) Perthes disease according to various classifications and grading schemes, and to compare the results to those achieved using other methods of treating Perthes disease reported in the literature.

## Materials and methods

Between July 2005 and December 2011, 23 patients with unilateral Legg–Calvé–Perthes disease were treated using a proximal femoral varus osteotomy procedure at Zagazig University Hospitals. The right side was affected in 15 patients and the left side in the remaining 8 patients. The mean age of the patients was 7.8 years (range: 6–11.5 years). All of them were males. The average follow-up was 36.2 months (range: 29–48 months). The patients’ main complaints were hip pain with limping and accompanying knee pain. The clinical complaints and their onset dates were noted, and the flexion, extension, abduction, adduction, and internal and external rotation ranges of the hips and limbs were also recorded. Clinical measurements and scanograms were used to measure and detect leg length discrepancies. Anteroposterior pelvic X-rays were obtained in neutral, abduction, and abduction–internal rotation positions as well as frog-leg lateral (Lauenstein) views for all of the patients. Epiphyseal or femoral head involvement was graded according to the classifications of Catterall [7] and Herring et al. [16]. Both the extrusion index [12] and Wiberg’s CE angle [40] were measured and graded, and the risk factors of the patients were also identified.

### Statistical analysis

The solftware SPSS version 16.0 for Windows (SPSS, Chicago, IL, USA, 2007) was used for statistical analysis. Wilcoxon’s signed-rank test was applied to the results and *p* > 0.05 was considered to be significant.

### Operative technique

General anesthesia was used for all patients. The patients were positioned in the supine position on a radiolucent orthopedic table. A lateral surgical approach to the proximal femur with an open-wedge subtrochanteric varus osteotomy was used. The osteotomy was fixed with a dynamic compression plate pre-bent from 15° to 20° varus and screws; the plate was (narrow) in 18 patients and broad in 5 patients (the width was varied according to the size of the patient). Immobilization for 6 weeks in a resin hip spica cast was employed for children from 6 to 7 years old, but the author preferred to use the less cumbersome short leg anti-rotation resin cast to prevent rotation for 3 weeks in patients >7 years old. Weight-bearing was avoided until mature bone was seen. Postoperatively, patients were followed up monthly for 1 year and then every 3 months after 1 year. The results of the treatment were evaluated according to the Iowa hip rating scale [23] as well as the measured amount of shortening in the extremity. The implants were removed after 12 months.

## Results

**Fig. 1:**
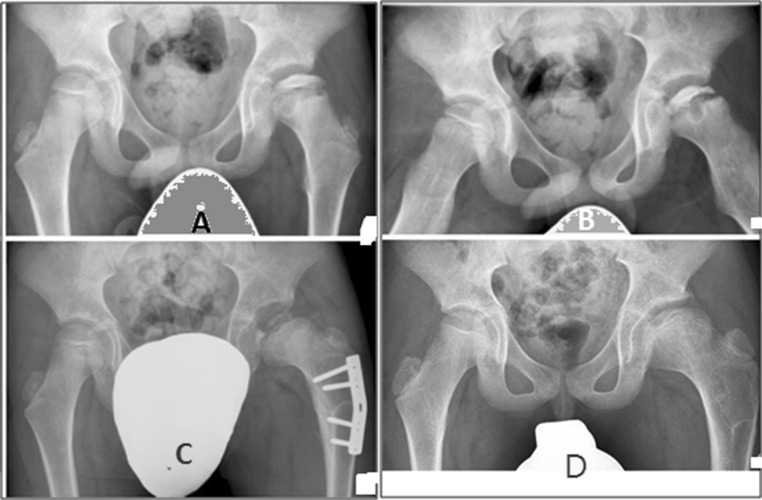
Radiographs show the hips of a 7.5-year-old boy with LCPD of the left hip (Catterall class IV, lateral pillar group C). He had symptoms for 5 months before diagnosis. **a** Preoperative AP radiograph; the preoperative epiphyseal extrusion index was 77.8. **b** Preoperative radiograph in Lauenstein projection shows reduced FHC. **c** Radiograph taken 12 months postoperatively shows containment after subtrochanteric femoral osteotomy with 25° varization and no rotation; epiphyseal extrusion improved to 29.1 postoperatively. **d** Radiograph taken at 48 months postoperatively shows an enlarged but spherical femoral head

Based on the Catterall and Herring classifications, the 23 patients were categorized as follows: 6 patients were Catterall group III (Herring group B), another 10 patients were Catterall group III (Herring group BC), and the other 7 patients were Catterall group IV (Herring group C). The preoperative and postoperative mean epiphyseal extrusion indices were as follows: group III (B), 10.88 % and 7.22 %, *P* = 0.027; group III (BC), 15.81 and 8.93 %, *P* = 0.005; group IV (C), 72.64 and 39.44 %, *P* = 0.018. The preoperative and postoperative mean Wiberg’s CE angle were as follows: group III (B), 26.88° and 37.81°, *P* = 0.028; group III (BC), 24.4° and 32.2°, *P* = 0.005; group IV (C), 20.89° and 28.41°, *P* = 0.018. The changes in the femoral neck–shaft angle were as follows: group III (B), 137.07° to 117.02°, *P* = 0.028; group III (BC), 137.0° to 117.3°, *P* = 0.005; group IV (C), 137.67° to 115.64°, *P* = 0.017. The preoperative and the postoperative mean Iowa clinical hip score were as follows: group III (B), 54.8 and 92.33, *P* = 0.027; group III (BC), 47.3 and 87.8, *P* = 0.005; group IV (C), 34.43 and 68.29, *P* = 0.017. Mean limb length discrepancy was 0.9 cm (range: 0.0–2 cm) of shortening at the operated side compared to the normal side at the last follow-up after plate removal. We did not see any progressive change in this parameter during the follow-up period, especially after hardware removal and in the younger boys. Limping related to limb length discrepancy and the gluteal weakness was reported for all patients, but this improved gradually over the course of 8 months after the operation. All of the osteotomies united within 3 months without loss of fixation (see Table 1; Fig. 1a–d).

**Table 1:** Pre- and postoperative results for the 23 patients with unilateral Legg–Calvé–Perthes disease who were treated using a proximal femoral varus osteotomy procedure

Patient no.	Age	Catt. class	Herr. class	Pre. op. EEI	Post. op. EEI	Pre. op. WCEA	Post. op. WCEA	Pre. op. NSA	Post. op. NSA	Pre. op. LHS	Post. op. LHS
1	6	III	B	10.6	7.1	25.1	38.1	140	119.5	57	94
2	6	III	B	10.3	7.2	30.3	39	139	118.7	41	90
3	7	III	B	10.4	7.3	30.1	38.6	137.2	115.6	60	94
4	7	III	B	10.5	7.1	30.2	38.2	137	116.1	60	94
5	9	III	B	11.4	7.4	25.2	35.4	133.2	115	54	93
6	9	III	B	12.1	7.2	20.4	37.6	136	117.2	57	89
Mean				10.88	7.22	26.88	37.81	137.07	117.02	54.8	92.33
Significance (*S*)				0.027		0.028		0.028		0.027	
7	6	III	BC	13.1	9.2	22	33.1	139	119.8	43	82
8	6	III	BC	17.2	10.2	27	34	138.5	118	48	91
9	6	III	BC	15.5	7.2	25	32.1	136	115.2	46	78
10	6.5	III	BC	17.6	8.1	28.3	33	138	118.3	44	88
11	6.5	III	BC	13.5	9.5	23	32.1	136.7	118	55	90
12	6.5	III	BC	13.5	7.3	23.4	30.3	138	116.8	44	92
13	7	III	BC	14.2	9.1	20.5	33.2	136.1	115.1	50	90
14	7	III	BC	16.3	9.5	25.4	31.4	140	119	56	87
15	8	III	BC	18.1	11.1	23.3	32.1	134	117	42	94
16	8	III	BC	19.1	8.1	26.3	31.1	134	115.6	45	86
Mean				15.81	8.93	24.4	32.2	137.0	117.3	47.3	87.8
Significance (*S*)				0.005		0.005		0.005		0.005	
17	11	IV	C	60.2	40.1	21	29.5	138.1	116	35	44
18	11	IV	C	70.3	50.2	20.2	27.2	137.5	110	31	40
19	11.5	IV	C	80.3	75.1	20.1	27.8	138.1	118	32	41
20	10	IV	C	79.1	26.1	21.1	26.1	136	115	34	90
21	9	IV	C	75.2	27.2	22.2	27.2	138	117	36	87
22	7.5	IV	C	77.8	29.1	20.1	30.6	138.5	117	37	86
23	7	IV	C	65.6	28.3	21.5	30.5	137.5	116.5	36	90
Mean				72.64	39.44	20.89	28.41	137.67	115.64	34.43	68.29
Significance (*S*)				0.018		0.018		0.017		0.017	

Mean patient age: 7.8 years old; age range: 6–11.5 years old

*Patient no* patient number, *Catt. class* Catterall classification, *Herr. class* Herring classification, *Pre. op. EEI* preoperative epiphyseal extrusion index, *Post. op. EEI* postoperative epiphyseal extrusion index, *Pre. op. WCEA* preoperative Wiberg’s central edge angle, *Post. op. WCEA* postoperative Wiberg’s central edge angle, *Pre. op. NSA* preoperative neck–shaft angle, *Post. op. NSA* postoperative neck–shaft angle, *Pre. op. LHS* preoperative Larson (Iowa) hip score, *Post. op. LHS* postoperative Larson (Iowa) hip score, *S* significant, *Ns* nonsignificant

## Discussion

The early goal of treatment is to prevent head deformation by weight-related forces during remodeling and ossification, so containment is the widely accepted treatment principle [28]. The main indication for operative containment treatment of Perthes disease is age >6 years along with lateral subluxation and advanced femoral head involvement [3, 14]. The most commonly reported surgical method for the treatment of Legg–Calvé–Perthes disease is proximal femoral varus osteotomy, which was first introduced in 1965 and has since become a popular surgical treatment for LCPD. Femoral varus osteotomy improves the intraosseous circulation, the mechanics around the proximal femoral head, and subsequently the degree of femoral head sphericity after healing, and it permits the regeneration of the necrotic tissues of the femoral head. It also prevents the subluxation of the femoral head, covering it with the acetabulum. It restores joint congruity and reduces femoroacetabular impingement [3–5, 13, 24, 28, 38].

On the other hand, the main aims of femoral valgus osteotomy are to reduce hinged abduction during remodeling and to improve the symptoms and the range of motion [20]. In severely deformed femoral heads treated with femoral valgus osteotomy, greater congruency is obtained in adduction rather than in abduction. Kim et al. evaluated the effectiveness of valgus osteotomy based on femoral head roundness, femoral head subluxation, and function. They found that this technique helped to keep the deformed femoral head inside the acetabulum during the fragmentation phase so that it could be remodeled to fit neatly inside the acetabulum [20]. Besides this, valgus osteotomy is valuable for relieving hinged abduction after skeletal maturity has been reached [41].

Recently, many authors have argued against nonoperative treatment, especially in children >6 years old with lateral pillar type B, B/C, or C LCPD. This group benefits more from varus or innominate osteotomy than nonoperative treatment because both pain and hip dysfunction are common in them. The clinical signs of femoroacetabular impingement and the radiographic signs of hip osteoarthritis were also found to be correlated with pain in nonoperatively treated patients [13, 22, 24, 33]. The main advantage of Salter or innominate osteotomy is its effect on femoral head remodeling during the remaining growth. Patients who are indicated for this osteotomy alone are usually younger children with a recent clinical onset of LCPD and no femoral head deformity or subluxation [34, 39].

One of the surgical methods used when other treatment options are contraindicated is arthrodiastasis of the hip joint with soft-tissue release. The advantages of this procedure are that it improves the range of motion, reduces superior and lateral subluxation, and provides better radiographic sphericity of the femoral head. This treatment can even be performed with distraction in stiff hips and deformed hips [1, 2, 21, 27]. In a comparative study by Voplon in which he used arthrodistraction as a primary treatment for active forms of LCP disease and prospectively compared the results with those obtained using Salter innominate osteotomy, although the methods gave similar final radiological results, morbidity was higher with arthrodistraction than with innominate osteotomy. Consequently, the author does not recommend arthrodistraction as a primary treatment for the early stages of Legg–Calvé–Perthes disease [39].

Although shelf acetabuloplasty leads to improved femoral head coverage, the available literature does not support the use of this procedure to prevent late osteoarthritis and improve function long term [17]. Recently, a new labral support technique has been reported. This shelf arthroplasty technique includes a minimal-incision variant of labral support shelf arthroplasty, arthroscopic visualization, and an allograft buttress on the shelf support that has been proposed to maintain containment. This minimal-incision technique yielded similar results to those obtained using a Petrie cast, a femoral varus osteotomy, or an innominate (Salter) osteotomy. The authors concluded that the labral support shelf arthroplasty technique is simple to perform and does not induce a permanent deformity in the proximal femur or acetabulum [6]. An advantage compared to femoral varus osteotomy is that there is additional lateral growth of the true acetabulum to generate more coverage following surgery. In this way, the labral support technique can stimulate lateral acetabular growth, restore the shelf after femoral epiphyseal reossification, and prevent subluxation [10].

Triple innominate osteotomy is considered one of the most efficient techniques for femoral head containment in any LCPD case. The main disadvantage of this technique is overcoverage of the femoral head, which can lead to pincer impingement. To prevent this complication, correction of the center-edge angle beyond 44° should be avoided [16].

Impingement and instability with intra- or extra-articular deformities of the hip can lead to joint damage and premature osteoarthritis of the hip. Surgical dislocation of the hip with trochanteric advancement faciliates lengthening of the femoral neck and the reduction of femoral head deformities. Leuing and Ganz reported 14 patients with surgical dislocation of the hip and trochanteric advancement with a minimum follow-up of 3 years. They found that pain, hip mobility, and gait improved greatly in this group of patients, with no major complications observed [25]. They noted that surgical dislocation of the hip yielded promising results in the treatment of femoral head deformities following LCPD [25, 31]. The authors reported transtrochanteric rotational osteotomy as a new technique for patients in whom the onset of LCPD occurs after 9 years of age. They concluded that this technique is an effective procedure for salvaging late-onset LCPD in affected hips, and that the amount of head involvement and the lateral pillar influence the surgical outcome [29, 36].

Herring et al. [14], in a prospective multicenter study, found a strong correlation between the lateral pillar classification, age at disease onset, and outcome in patients with Legg–Calvé–Perthes disease. Patients >8.0 years old at disease onset who had a hip categorized as lateral pillar B or B/C according to the Herring classification were found to have better outcomes following surgical treatment with either femoral varus osteotomy or innominate osteotomy than they did with nonoperative treatment. Group B hips in children <8.0 years of age at disease onset have very favorable outcomes that are unrelated to choice of treatment, whereas group C hips in children of all ages frequently have poor outcomes, which again appear to be unrelated to the choice of treatment.

In contrast to Herring, in another multicenter Norwegian prospective study on Legg–Calvé–Perthes disease that was published by Terjesen et al. [37], 70 patients who were diagnosed at >6 years old with unilateral LCPD and had femoral head necrosis of more than 50 % were treated with femoral varus osteotomy. In that study, both the Catterall and Herring classifications of necrosis were applied, and the results were compared with a control group of 51 similar children who received physiotherapy treatment. They concluded that, in children aged 6.0–10.0 years in whom the whole femoral head is affected, femoral head sphericity at 5 years follow-up after femoral varus osteotomy was better than that achieved with physiotherapy. Those results are in good accord with the results of the present study because the three unsuccessfully treated patients in the present study were classified as having Catterall group IV (Herring type C) LCPD and were over 10 years old. These findings support the efficacy of early surgery for appropriately selected patients due to the remarkable decrease in the ability to remodel after 5–6 years of age, meaning that patients over 6 years of age should be the main candidates for operative treatment [3, 18, 19, 37].

Rather than Salter’s osteotomy, the author of the present study preferred proximal femoral varus osteotomy because this method achieves decompression, enables dynamic treatment, does not increase intra-articular pressure, and does not cause a frozen joint postoperatively with good coverage of the femoral head in the hip joint [3, 12, 15, 30, 37]. It is also worth noting that femoral varus osteotomy is said to have certain disadvantages or complications, such as femoral shortening, limping, excessive varus, nonunion, and overgrowth and elevation of the greater trochanter [3, 12, 15, 30, 37]. The most important predictor of leg length discrepancy (LLD) is the extent of lateral pillar involvement, and no other factor (including age, sex, and treatment modality) is correlated with LLD at skeletal maturity [32]. In the present study, the author decided to use an open-wedge osteotomy, as persistent limb shortening tends to be greater after a closed-wedge osteotomy in the older child. We did not see any progressive change in this parameter during the follow-up period, especially after hardware removal and in the younger boys, but it may decrease with time as the varus angulation of the subtrochanteric osteotomy gradually changes.

Limping related to limb length discrepancy or gluteal weakness or both have generally been reported by other authors after a proximal femoral varus osteotomy. In the present study, the author encountered limping, but the limping gradually improved within 8 months after the operation, in agreement with observations reported by other authors. No complications such as delayed union, nonunion, overgrowth, or elevation of the greater trochanter were encountered in the present study. However, the author did face common problems and limitations associated with studies in this field, including the variable nature of Perthes disease (which makes the condition difficult to study) and the use of different classification systems and outcome measures (which leads to confusion). Analysis of surgical procedures is hampered by the use of small subject groups, the infrequent use of a control group, the unmatched selection of patients of varying ages, and the varying severity of the disease process.

In conclusion, proximal femoral varus osteotomy gives good results in children aged 6–10 years who do not exhibit any femoral head deformity or flattening, especially those with good containment in abduction. Treatment failure is not usually because of the treatment method; it is due to technical errors, inappropriate patient selection, and delayed treatment. All recently reported techniques aim to reshape the femoral head in both congruency and size to match the acetabulum and sequentially decrease the impingement, as well as to restore the normal cartilage in the weight-bearing zone of the head.

## References

[1] Aldegheri R, Trivella G, Saleh M (1994). Articulated distraction of the hip: conservative surgery for arthritis in young patients. Clin Orthop Relat Res.

[2] Aldegheri R, Trivella G, Saleh M, de Bastiani G, Apley G, Goldberg A (2000). Articulated distraction of the hip. Orthofix external fixation in trauma and orthopaedics.

[3] Atlihan D, Mehmet S, Yildirim H (1999). Proximal femoral varus osteotomy for Perthes disease. Turk J Arthroplast Arthrosc Surg.

[4] Axer A, Gershuni DH, Hendel D (1980) Indications for femoral osteotomy in Legg–Calvé–Perthes disease. Clin Orthop 150:78–87 7428247

[5] Axer A (1965) Subtrochanteric osteotomy in the treatment of Legg–Calvé–Perthes disease. JBJS Br 47:489–499 14341066

[6] Bowen JR, Guille JT, Jeong C, Worananarat P, Oh CW, Rodriquez A, Holmes L, Rogers KJ (2011) Labral support shelf arthroplasty for containment in early stages of Legg–Calvé–Perthes disease. J Pediatr Orthop 31:S206–S211 10.1097/BPO.0b013e31822910ba 21857440

[7] Catterall A (1982) Legg–Calvé–Perthes disease. Churchill Livingstone, New York, pp 8–33, 81–109

[8] Choi IH, Yoo WJ, Cho TJ, Moon HJ (2011) The role of valgus osteotomy in LCPD. J Pediatr Orthop 31:ss217–ss222 10.1097/BPO.0b013e318223b404 21857442

[9] Cooperman DR, Stulberg SD (1986). Ambulatory containment treatment in Perthes’ disease. Clin Orthop Relat Res.

[10] Domzalski ME, Glutting J, Bowen JR, Littleton AG (2006) Lateral acetabular growth stimulation following a labral support procedure in Legg–Calvé–Perthes disease. J Bone Joint Surg Am 88:1458–1466 10.2106/JBJS.E.00689 16818970

[11] Fulford GE, Lunn PG, Macnicol MF (1993) A prospective study of nonoperative and operative management for Perthes’ disease. J Pediatr Orthop 13:281–285 10.1097/01241398-199305000-00001 8496357

[12] Green NE, Beauchamp RD, Griffin PP (1981) Epiphyseal extrusion as a prognostic index in Legg–Calvé–Perthes disease. JBJS Am 63(6):900–905 7240330

[13] Heikkinen SE, Puranens J, Suramo I (1976) The effect of intertrochanteric osteotomy on the venous drainage of the femoral neck in Perthes disease. Acta Orthop Scand 47:89–95 10.3109/17453677608998978 1266598

[14] Herring JA, Kim HT, Browne R (2004) Legg–Calvé–Perthes disease. Part II. Prospective multicenter study of the effect of treatment on outcome. JBJS Am 86(10):2121–2134 15466720

[15] Herring JA, Neustadt JB, Williams JJ, Early JS, Browne RH (1992). The lateral pillar classification of Legg–Calvé–Perthes disease. J Pediatr Orthop.

[16] Hosalkar H, Munhoz da Cunha AL, Baldwin K, Ziebarth K, Wenger DR (2012) Triple innominate osteotomy for Legg–Calvé–Perthes disease in children: does the lateral coverage change with time? Clin Orthop Relat Res 470:2402–2410 10.1007/s11999-011-2189-z PMC3830082 22125244

[17] Hsu JE, Baldwin KD, Tannast M, Hosalkar H (2012) What is the evidence supporting the prevention of osteoarthritis and improved femoral coverage after shelf procedure for Legg–Calvé–Perthes disease? Clin Orthop Relat Res 470:2421–2430 10.1007/s11999-011-2220-4 PMC3830099 22194022

[18] Joseph B, Price TC (2011) Principles of containment treatment aimed at preventing femoral head deformation in Perthes disease. Orthop Clin N Am 42:317–327 10.1016/j.ocl.2011.04.001 21742143

[19] Joseph B, Srinivas G, Thomas R (1996) Management of Perthes disease of late onset in southern India. The evaluation of a surgical method. JBJS Br 78(4):625–630 8682832

[20] Kim HT, Gu JK, Bae SH, Jang JH, Lee JS (2013) Does valgus femoral osteotomy improve femoral head roundness in severe Legg–Calvé–Perthes disease? Clin Orthop Relat Res 471:1021–1027 10.1007/s11999-012-2606-y PMC3563831 23096935

[21] Laklouk MA, Hosny GA (2012). Hinged distraction of the hip joint in the treatment of Perthes disease: evaluation at skeletal maturity. J Pediatr Orthop B.

[22] Larson AN, Sucato DJ, Herring JA, Adolfsen SE, Kelly DM, Martus JE et al (2012) A prospective multicenter study of Legg–Calvé–Perthes disease: functional and radiographic outcomes of nonoperative treatment at a mean follow-up of twenty years. J Bone Joint Surg Am 94:584–592 10.2106/JBJS.J.01073 22488614

[23] Larson CB (1963). Rating scale for hip disabilities. Clin Orthop Relat Res.

[24] Lee DY, Seang SC, Chai IH, Chung CY, Chang BS (1992) Changes of blood flow of the femoral head after subtrochanteric osteotomy in Legg–Calvé–Perthes disease: a serial scintigraphic study. J Pediatr Orthop 12:731–734 10.1097/01241398-199211000-00006 1452741

[25] Leunig M, Ganz R (2011) Relative neck lengthening and intracapital osteotomy for severe Perthes and Perthes-like deformities. Bull NYU Hosp Jt Dis 69(Suppl 1s):s62–s67 22035488

[26] MacEwen GD (1985) Conservative treatment of Legg–Calvé–Perthes disease condition. In: Fitzgerald RH Jr (ed) The hip: proceedings of the Thirteenth Open Scientific Meeting of the Hip Society. CV Mosby, St. Louis, pp 17–23

[27] Maxwell SL, Lappin KJ, Kealey WD, McDowell BC, Cosgrove AP (2004). Arthrodiastasis in Perthes’ disease. JBJS Br.

[28] Mazloumi SM, Ebrahimzadeh MH, Kachooei AR (2014) Evolution in diagnosis and treatment of Legg–Calvé–Perthes disease. Arch Bone Jt Surg 2(2):86–92 **(Epub 2014 Jun 15)** PMC4151449 25207324

[29] Nakashima Y, Kubota H, Yamamoto T, Mawatari T, Motomura G, Iwamoto Y (2011) Transtro-chanteric rotational osteotomy for late-onset Legg–Calvé–Perthes disease. J Pediatr Orthop 31:s223–s228 10.1097/BPO.0b013e318223b4f3 21857443

[30] Noonan KJ, Price CT, Kupiszewski SJ, Pyevich M (2001). Results of femoral varus osteotomy in children older than 9 years of age with Perthes disease. J Pediatr Orthop.

[31] Novais EN (2013) Application of the surgical dislocation approach to residual hip deformity secondary to Legg–Calvé–Perthes disease. J Pediatr Orthop 33:s6209 10.1097/BPO.0b013e318281132d 23764795

[32] Park KW, Jang KS, Song HR (2013) Can residual leg shortening be predicted in patients with Legg–Calvé–Perthes disease? Clin Orthop Relat Res 471:2570–2577 10.1007/s11999-013-3009-4 PMC3705048 23616268

[33] Purvis JM, Dimon JH 3rd, Meehan PL, Lovell WW (1980) Preliminary experience with the Scottish Rite Hospital abduction orthosis for Legg–Perthes disease. Clin Orthop 150:49–53 7428242

[34] Saran N, Varghese R, Mulpuri K (2012) Do femoral or salter innominate osteotomies improve fe-moral head sphericity in Legg–Calvé–Perthes disease? A meta-analysis. Clin Orthop Relat Res 470:2383–2393 10.1007/s11999-012-2326-3 PMC3830109 22467420

[35] Sponseller PD, Desai SS, Millis MB (1988) Comparison of femoral and innominate osteotomies for the treatment of Legg–Calvé–Perthes disease. JBJS 70-A(8):1131–1139 3417698

[36] Sucato DJ (2013). Role of femoral head surgery in skeletally mature Perthes disease. J Pediatr Orthop.

[37] Terjesen T, Wiig O, Svenningsen S (2012) Varus femoral osteotomy improves sphericity of the femoral head in older children with severe form of Legg–Calvé–Perthes disease. Clin Orthop Relat Res 470:2394–2401 10.1007/s11999-011-2181-7 PMC3830087 22101403

[38] Thompson GH (2011) Salter osteotomy in Legg–Calvé–Perthes disease. J Pediatr Orthop 31:S192–S197 10.1097/BPO.0b013e318223b59d 21857438

[39] Volpon JB (2012) Comparison between innominate osteotomy and arthrodistraction as a primary treatment for Legg–Calvé–Perthes disease: a prospective controlled trial. Int Orthop 36(9):1899–1905. doi:10.1007/s00264-012-1598-2**(Epub 2012 Jul 19)** 10.1007/s00264-012-1598-2 PMC3427447 22810494

[40] Wiberg G (1953). Shelf operation in congenital dysplasia of the acetabulum and in sublaxation and dislocation of the hip. JBJS Am.

[41] Yoo WJ, Choi IH, Moon HJ, Chang S, Cho TJ, Choi YH et al (2013) Valgus femoral osteotomy for non containable Perthes hips: prognostic factors of remodeling. J Pediatr Orthop 33:650–655 10.1097/BPO.0b013e31829569c8 23812133

